# Overcoming drug resistance through extracellular vesicle-based drug delivery system in cancer treatment

**DOI:** 10.20517/cdr.2024.107

**Published:** 2024-12-12

**Authors:** Long Zheng, Ruibai Chang, Bingjing Liang, Yitong Wang, Yushan Zhu, Zijing Jia, Jindian Fan, Zhe Zhang, Bo Du, Dexin Kong

**Affiliations:** ^1^College of Chinese medicine, Tianjin University of Traditional Chinese Medicine, Tianjin 301617, China.; ^2^Tianjin Key Laboratory of Technologies Enabling Development of Clinical Therapeutics and Diagnostics, School of Pharmaceutical Sciences; Tianjin Medical University, Tianjin 300070, China.; ^3^Key Laboratory of Immune Microenvironment and Diseases (Ministry of Education), Tianjin Medical University, Tianjin 300070, China.; ^4^Tianjin Key Laboratory of Biomedical Materials, Biomedical Barriers Research Center, Institute of Biomedical Engineering, Chinese Academy of Medical Sciences & Peking Union Medical College, Tianjin 300192, China.; ^#^Authors contributed equally.

**Keywords:** Extracellular vesicles, drug delivery systems, drug resistance, tumor microenvironment, cancer therapy

## Abstract

Drug resistance is a major challenge in cancer therapy that often leads to treatment failure and disease relapse. Despite advancements in chemotherapeutic agents and targeted therapies, cancers often develop drug resistance, making these treatments ineffective. Extracellular vesicles (EVs) have gained attention for their potential applications in drug delivery because of their natural origin, biocompatibility, and ability to cross biological barriers. Using the unique properties of EVs could enhance drug accumulation at target sites, minimize systemic toxicity, and precisely target specific cells. Here, we discuss the characteristics and functionalization of EVs, the mechanisms of drug resistance, and the applications of engineered EVs to overcome drug resistance. This review provides a comprehensive overview of the advancements in EV-based drug delivery systems and their applications in overcoming cancer drug resistance. We highlight the potential of EV-based drug delivery systems to revolutionize cancer therapy and offer promising strategies for more effective treatment modalities.

## INTRODUCTION

Drug resistance remains a formidable obstacle in cancer therapy, often leading to treatment failure and disease relapse^[1,2]^. Despite the advancements in the development of chemotherapeutic agents and targeted therapies, cancers often develop resistance mechanisms that render these treatments ineffective^[3]^. The emergence of drug-resistant cancer cells can be attributed to various factors including genetic mutations, altered drug metabolism, efflux pump overexpression, and modifications in cell signaling pathways^[4-6]^. These complexities necessitate innovative approaches for enhancing the efficacy of cancer treatments.

Extracellular vesicles (EVs), including exosomes, microvesicles, and apoptotic bodies, have garnered considerable attention in recent years because of their potential role in intercellular communication and ability to transfer bioactive molecules between cells^[7-11]^. EVs are lipid bilayer-enclosed particles released by cells that carry diverse cargoes such as proteins, lipids, nucleic acids, and other small molecules^[12-14]^. Their natural origin, biocompatibility, and inherent ability to cross biological barriers make them promising candidates for drug delivery^[15,16]^.

Recent studies have highlighted the potential of EV-based drug delivery systems to overcome drug resistance in cancer^[7,13,17]^. By leveraging their unique properties, EVs can be engineered to directly deliver chemotherapeutic agents, small interfering RNAs (siRNAs), microRNAs (miRNAs), and other therapeutic molecules to cancer cells, thereby enhancing drug accumulation at target sites and minimizing systemic toxicity^[18-20]^. In addition, EVs can be modified to target specific cell types, thereby providing a level of precision that conventional drug delivery methods lack^[21-23]^.

This review aims to provide a comprehensive overview of the current advancements in EV-based drug delivery systems and their applications in overcoming drug resistance in cancer. We discuss EVs and their functionalization, mechanisms of drug resistance in cancer, and the application of engineered EVs to overcome drug resistance. By exploring these aspects, we hope to shed light on the potential of EV-based drug delivery systems to revolutionize cancer therapy and address the pressing issue of drug resistance in cancer.

## EVS AND THEIR FUNCTIONALIZATION

### EVs

**Figure 1:**
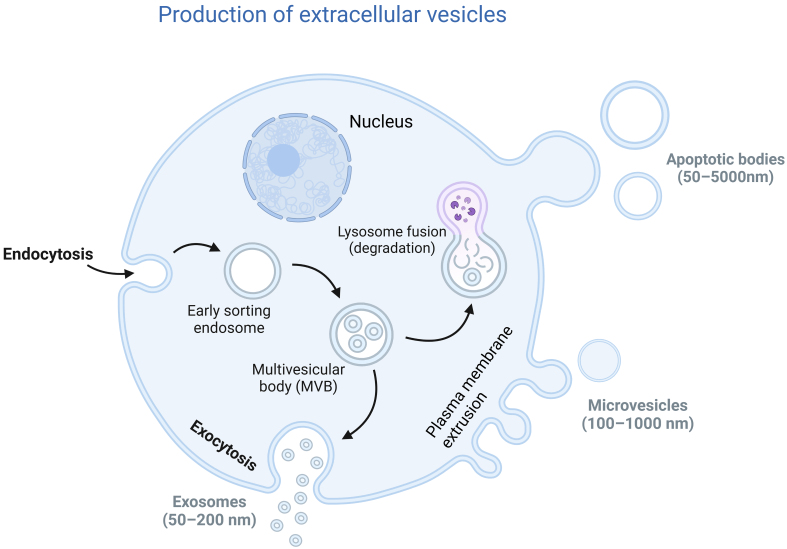
Schematic representation of subtypes of EVs. EVs: Extracellular vesicles.

In 1985, Pan *et al*. observed the vesicle structures secreted during the maturation of reticulocytes using a microscope^[24]^. Over the past few decades, in-depth studies have provided a clearer understanding of the biological processes and structure of EVs. The 2023 edition of the International Society for EVs provides a clear definition. The term “Evs” refers to particles that are released from cells, delimited by a lipid bilayer, and cannot replicate on their own (that is, they do not contain a functional nucleus)^[8]^. EVs are classified into three subtypes based on their size, structure, and biological processes of formation: exosomes, microvesicles, and apoptotic bodies [Figure 1]^[9]^. Exosomes are vesicles formed by the inward budding of the membrane through endocytosis and then secreted outside the cell. Microvesicles are formed by the outward budding and shedding of the cell membrane; apoptotic bodies are produced by the disintegration of cells during programmed cell death^[10,11]^. EVs act as messengers for intercellular communication, with numerous studies indicating that they play significant roles in tumor formation and development and are closely related to cancer resistance mechanisms^[7]^. In addition, EVs serve as excellent drug carriers, capable of delivering proteins, DNA, RNA, and other molecules for the treatment of cancer or chronic wounds^[12-14,25]^.

### Characterization of EVs

EVs are complex structures containing various proteins and lipids. Common laboratory characterization methods for studying EV structures include nanoparticle tracking analysis (NTA), dynamic light scattering (DLS), transmission electron microscopy (TEM), flow cytometry, and western blotting (WB).

NTA uses laser scattering and the principle of the random Brownian motion of particles to measure the size distribution and concentration of EVs in liquid suspensions, making it one of the most used characterization methods in EV research^[26,27]^. DLS, similar in principle to NTA, also detects the size of EVs but measures the overall scattering intensity from the entire sample^[28,29]^. TEM enables direct observation of EV morphological characteristics^[30]^. Flow cytometry can be used to quantitatively analyze EVs by detecting the signal intensity of fluorescence-labeled antibodies specific to membrane surface markers^[31-33]^. WB is commonly used to detect proteins within EVs, which are first separated by gel electrophoresis and labeled with antibodies for qualitative or semi-quantitative analysis of the labeled proteins^[8,34]^.

### Isolation of EVs

EVs are small vesicles of varying sizes that are secreted by cells and are characterized by low toxicity, low immunogenicity, and excellent biocompatibility, making them ideal drug delivery vehicles. The following sections outline several commonly used methods for isolating EVs from cells. Different EV isolation techniques of EVs are summarized in Table 1.

**Table 1:** Comparison of different isolation methods of EVs

**Isolation method**	**Principle**	**Advantages**	**Disadvantages**	**Application**	**Ref.**
Ultracentrifugation	Isolation based on size and density through centrifugal force to separate the impurities from samples	High productivity; Simple procedure; Easy to use	Time-consuming; Damage to the EVs; Formation of aggregates and precipitation	Blood, urine, cerebrospinal fluid	[35-40]
Ultrafiltration	Isolation uses a porous filter of size‐exclusion limits, which removes smaller molecules (non‐EV) by flow‐through	Simple procedure; Suitable for large-volume isolation; Retain EVs functionality and integrity	Time-consuming; Low purity; Low productivity	Blood, urine, and saliva	[41-44]
Precipitation	Induced clumping of EVs in the form of pellet by decreasing the solubility	Simple procedure; No additional equipment required; Suitable for large-volume isolation	Low efficiency; Time-consuming; High cost; Low purity with co-precipitation of protein and other molecules	Plasma, urine, saliva	[45-47]
Size-exclusion chromatography	Isolation based on single isolation column containing porous beads with radii smaller than EVs	High purity; High homogeneity; Retain EVs functionality and integrity	Low productivity; Not suitable for higher sample volumes	Cell culture supernatants and complex biological fluids like plasma	[48-55]
Immunoaffinity	Using immunoaffinity substances such as proteins, aptamers, and antibodies that can recognize the surface-specific structure of EVs to recognize and capture EVs	High efficiency; High specificity and purity; Retain EVs functionality and integrity	Low productivity; Only enrichment of EVs with specific proteins; Not suitable for larger sample volumes; High cost	Tumor cells, immune cells	[56-59]

EVs: Extracellular vesicles.

#### Ultracentrifugation

Ultracentrifugation separates components in a solution based on size and density differences and is the most used method because of its simplicity^[35,38,39]^. EVs can be isolated from samples such as human urine, saliva, intestinal digestive fluid, cardiac and alveolar cell culture media, mouse hippocampal interstitial fluid, and milk using ultracentrifugation^[60-66]^. However, this method has several drawbacks, including time-consuming extraction and the low purity of isolated EVs, making it suitable only for small sample sizes^[36,40,67]^.

#### Precipitation

Precipitation is a straightforward, instrument-free technique for exosome isolation. It is a polymer-based method in which a sample is mixed with a polymer at a low temperature and the salt concentration is adjusted, inducing EV aggregation and precipitation by reducing solubility^[45-47]^. For example, Mishra *et al.* used polyethylene glycol (PEG) to precipitate EVs from the plasma of patients to achieve high purity^[68]^. Similarly, Pecksen *et al.* used PEG to isolate monocyte-derived EVs from a co-culture of induced pluripotent stem cells and human monocytes^[69]^. Matchett and Kornbluth used a modified PEG-acetate reagent to isolate natural killer (NK) cell-derived EVs^[70]^.

#### Size-exclusion chromatography

Size-exclusion chromatography (SEC) effectively separates EVs according to particle size, using porous beads that are smaller than EVs. It is suitable for isolating EVs from complex tissue or fluid samples and has significant clinical research applications^[49,50,52,53,55]^. For example, Livkisa *et al.* used SEC to remove impurities, such as carrier proteins, coagulation factors, and complement proteins, from platelet lysates, thereby achieving high-purity EVs^[71]^.

#### Immunoaffinity capture

Immunoaffinity capture uses substances such as proteins, aptamers, and antibodies that specifically recognize and capture EVs based on the unique structures on their surfaces. This method exhibits high efficiency, specificity, and purity^[56-59]^. For instance, Koksal *et al.* used phycoerythrin-conjugated human CD9, AFP, and GPC3 antibodies to capture exosomes from the sera of patients^[72]^. Wang *et al.* modified nanofibers with CD63 antibodies to effectively capture EVs expressing surface CD63 proteins^[73]^.

#### Ultrafiltration

The ultrafiltration technique uses a porous filter of a certain size with exclusion limits to eliminate smaller molecules (non-EVs) by flow-through^[74]^. This method is typically combined with other methods. Hanson *et al.* used ultrafiltration and SEC to isolate EVs from C2C12 myoblasts^[75]^. Moreover, ultrafiltration combined with differential centrifugation or ultracentrifugation can be used to isolate EVs derived from stem cells and gut bacteria^[76-78]^.

#### Other methods

Existing methods for separating EVs cannot meet the experimental requirements, and there is an urgent need for new separation methods or improvements to existing ones. Bathini *et al.* proposed a liquid biopsy chip using the synthetic peptide Vn96 for EV isolation based on magnetic particles^[79]^. Chattrairat *et al.* upgraded the conventional well plate assay to an all-in-one nanowire-integrated well plate assay for charge-based EV capture and membrane protein analysis^[80]^. Zhang *et al.* developed a rapid EV aggregation induced *in-situ* miRNA detection technology based on a cationic lipid-polymer hybrid nanoparticle encapsulating cascade system of catalytic hairpin assembly and CRISPR-Cas12a, achieving an enrichment efficiency of > 90% in 30 min, 5 times higher than that of ultracentrifugation^[81]^.

### Strategies to functionalize EVs

Methods for functionalizing EVs primarily include surface modification and loading of targeted drug molecules.

#### Surface modification strategies

**Figure 2:**
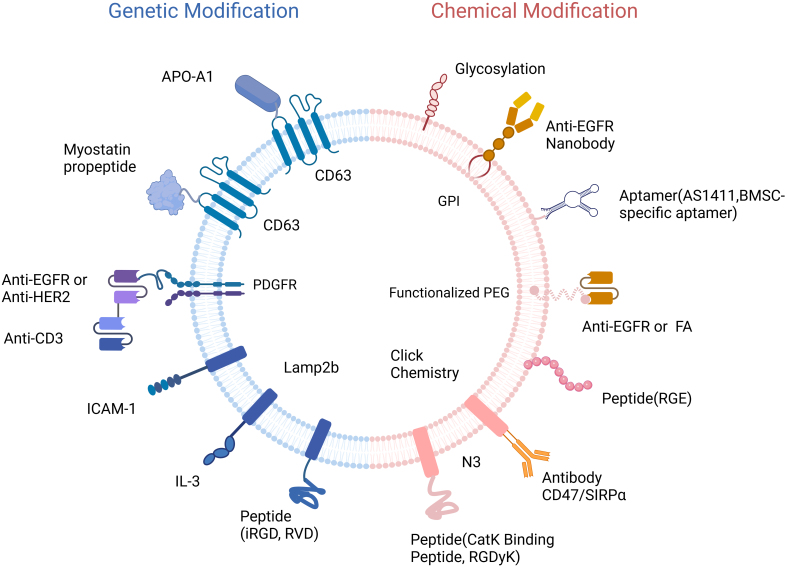
Alteration of EV surface by chemical modification and genetic engineering. EVs: Extracellular vesicles; EGFR: epidermal growth factor receptor; PDGFR: platelet-derived growth factor receptor; GPI: glycosylphosphatidylinositol.

Before using natural EVs for drug delivery, EV membranes can be modified with various molecules to increase the specificity for target cells. The surface modification strategies for EVs include genetic modification, chemical modification, metabolic labeling, enzymatic conjugation, membrane fusion with liposomes, and affinity binding^[82-85]^. Here, we focus on the current EV surface modification strategies, including genetic and chemical modifications [Figure 2].

Genetic modifications This approach enhances EV targeting to specific cellular and tissue environments but is limited to targeting genetically encodable motifs^[86]^. Genetic modifications involve the fusion of gene sequences of proteins or polypeptides with selected EV membrane protein gene sequences^[21]^ [Table 2].

**Table 2:** Applications of genetic modifications to EVs

**Fusion protein**	**EVs source**	**Modification details**	**Target**	**Comments**	**Ref.**
Lamp-2b	MSCs	IL3	LSCs	Enhanced bone marrow homing and selective targeting of LSCs	[22]
Lamp-2b	Dendritic cells	RVG peptide	Neurons, microglia, Oligodendrocytes	Demonstrates effective siRNA brain delivery	[87]
Lamp-2b	Mouse immature dendritic cells	iRGD peptide	αv integrin-positive breast cancer cells	Enhanced targeting ability	[88]
Lamp-2b	HEKT 293 cell	ICAM-1	Recombinant LFA-1	Interacts with T cells, efficiently delivers functional plasmids	[89]
PDGFR	Expi293F cells	CD3 and EGFR antibody	T cell CD3 and EGFR	Cross-links T cells and EGFR-positive cancer cells, triggers antitumor immunity	[90]
PDGFR	Expi293 cells	CD3 and HER2 antibodies	T cell CD3 and breast cancer-associated HER2 receptors	Promising exosome-based HER2 breast cancer immunotherapy	[91]
CD63	HEK293T cells	Apo-A1 complex	HepG2 cells	Shows effective targeted miRNA delivery for liver cancer therapy	[92]

EVs: Extracellular vesicles; MSCs: mesenchymal stem cells; EGFR: epidermal growth factor receptor; PDGFR: platelet-derived growth factor receptor; LSCs: leukemic stem cells.

Lamp2b, a member of the lysosome-associated membrane protein family, is highly expressed in EVs derived from dendritic cells (DCs) and is commonly used as a fusion protein for genetic modification of EVs. Fusing the Rabies Virus Glycoprotein-Derived Peptide with the Lamp2b on EV surfaces improves the brain-targeting ability of EVs, making them potential carriers for brain disease therapeutics^[87]^. The iRGD specifically binds to α_v_-integrins, and fusing it with Lamp2b on EV surfaces efficiently targets αv-integrin-positive breast cancer cells^[88]^. EVs overexpressing the fused protein Lamp2b-IL3 effectively target LSCs and are a potential drug carrier for acute myeloid leukemia^[22]^. Fusing Intercellular adhesion molecule-1 with Lamp2b on EVs can specifically target T cells^[89]^.

The platelet-derived growth factor receptor (PDGFR) is expressed on EV membranes and can be fused with various antibodies. For example, combining anti-CD3 and anti-epidermal growth factor receptor (EGFR) antibodies with the PDGFR region on EV membranes can simultaneously target T cells and EGFR-expressing triple-negative breast cancer cells^[90]^. Similarly, the fusion of anti-CD3 and anti-HER2 antibodies targets T cells and HER2-expressing breast cancer cells^[91]^.

The CD63, a common tetraspanin on EV surfaces, has been used for genetic modifications. The fusion of CD63 with the Apo-A1 sequence enables the targeting of HepG2 cells^[92]^. Furthermore, combining the inhibitory domain of the myostatin propeptide with CD63 can enhance the delivery and efficacy of the treatment for Duchenne muscular dystrophy^[93]^.

Chemical modifications Chemical modifications involve the attachment of specific molecules to the surface of EVs via chemical reactions^[94]^. These modifying molecules are primarily antibodies, peptides, or other targeting aptamers that selectively bind to receptors on cancer or immune cells to enhance their therapeutic effects^[95-97]^ [Table 3].

**Table 3:** Applications of chemical modifications to EVs

**EVs source**	**Modification details**	**Target**	**Comments**	**Ref.**
MSCs	c(RGDyK) peptide	The lesion region of the ischemic brain	Suppress the inflammatory response and cellular apoptosis in the lesion region	[98]
BM-MSCs	Cathepsin K	SMCs	Improve the cell-specific uptake of EVs and may be effective in facilitating AAA-targeted therapy	[99]
M1 macrophages	Antibodies of CD47 and SIRPα	aCD47 and CD47 on tumor cell surface	Actively target tumors through the specific recognition between aCD47 and CD47 on tumor cell surface	[100]
U-87 cells	DSPE-PEG2000-FA	U-87 cells	Reduce the side effects of TMZ (minimal change in body weight), prolong survival, and inhibit tumor growth in mouse glioma models	[101]
Neuro2A cells	Nanobody-PEG-lipids	EGFR	Improve cell specificity and prolong circulation time, potentially increasing; EV accumulation in targeted tissues and improving cargo delivery.	[102]
Neuro2A cells	GPI-linked EGFR nanobodies	EGFR	Greatly improve EV binding to tumour cells	[103]
Primary dendritic cells	Aptamer AS1411	MDA-MB-231 cells	Tumor targeting	[104]
Mouse liver proliferative cells	Glycosylation	-	Promote the accumulation of EVs in the lungs	[105]
M2 macrophages	BMSC-specific aptamer	BMSCs	promote the proliferation, migration, and osteogenic differentiation of BMSC	[106]
HeLa cells	pH-sensitive fusogenic peptide	-	The effective cellular uptake and cytosolic release of an artificially encapsulated dextran macromolecule (70 kDa) in exosomes	[107]
HEK 293 T cells	CPP-conjugated-lipids	HUVECs	Improve the intracellular delivery of exosomes	[108]
Raw264.7 cells	Neuropilin-1-targeted peptide	Glioma cells	cross the BBB, achieve targeted imaging and therapy of glioma	[23]

EVs: Extracellular vesicles; MSCs: mesenchymal stem cells; TMZ: temozolomide; PEG: polyethylene glycol; EGFR: epidermal growth factor receptor; GPI: glycosylphosphatidylinositol.

The lipid surfaces of EVs can be functionalized to improve their targeting capabilities by incorporating various small molecules using click chemistry. Using click chemistry, neuropilin-1 targeting peptides (RGERPPR, RGE) can be conjugated to the lipid surface of EVs, facilitating targeted delivery to glioma tissue^[23]^. There are reports where the parental cells can be metabolically engineered to secrete EVs with surface-modified azides. Click chemistry can be used to modify the azides on the surface of EVs with antibodies or peptides to enhance the targeting ability of EVs^[83]^. For instance, exosomes modified with c(RGDyK) peptides using click chemistry targeted ischemic brain regions in a mouse model of transient middle cerebral artery occlusion^[98]^. Cathepsin K (CatK), a cysteine protease overexpressed in the vascular walls of abdominal aortic aneurysms, can be targeted by attaching CatK-targeting peptides to EVs using click chemistry, thereby improving their uptake by tumor cells^[99]^. Similarly, azide-modified exosomes can be conjugated with dibenzocyclooctyne-modified antibodies of CD47 and SIRPα (aCD47 and aSIRPα) through a pH-sensitive linker. These exosomes can actively target tumors^[100]^.

Chemical modifications of EV membranes can also be achieved using other methods. Functionalized PEG fused with EV membrane can achieve different functional effects. For example, EVs combined with folic acid-modified functional PEG can effectively target glioma cells and deliver drugs^[101]^. EVs conjugated with EGFR antibody-modified functional PEG can target tumor cells overexpressing EGFR receptors^[102]^. Glycosylphosphatidylinositol (GPI) anchor proteins, commonly occurring on cell membranes and expressed on EV membranes, can bind to anti- EGFR nanobodies, enabling the targeting of tumor cells overexpressing EGFR^[103]^.

The EVs membrane can also bind to the aptamer AS1411 through hydrophobic interactions, allowing the targeting of breast cancer tissues^[104]^. In addition, chemical modifications can influence the affinity of EVs for specific tissues. For example, EVs modified with neuraminidase have a strong affinity for the liver, whereas EVs modified with glycosidase have a strong affinity for the lung tissue^[105]^.

#### Therapeutic cargo loading in engineered EVs

**Figure 3:**
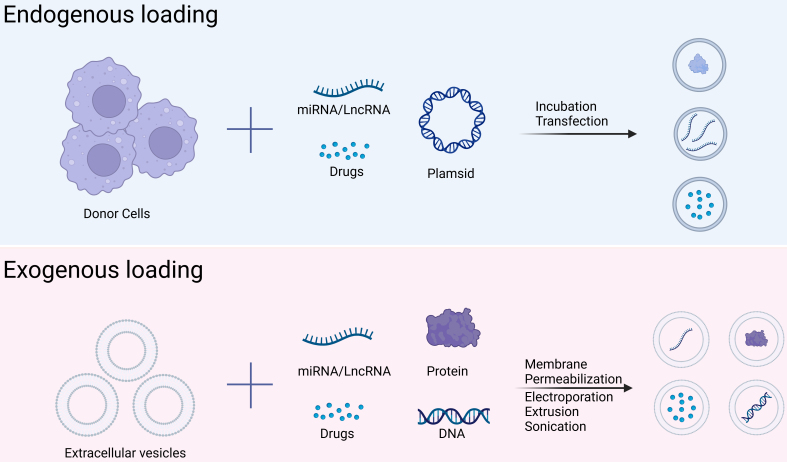
Therapeutic cargo loading methods in engineered EVs. Endogenous loading involves transfecting donor cells to express specific molecules or incubating them with drugs, enabling the cells to absorb the drugs and release drug-loaded extracellular vesicles. Exogenous loading involves isolating extracellular vesicles from cells and then using physical or chemical methods to load drugs into the vesicles. EVs: Extracellular vesicles.

Functionalized EVs not only require surface modifications to enhance targeting capabilities but also need to load drugs into the vesicles. The commonly used methods for drug loading are divided into two categories: endogenous and exogenous loading [Figure 3]. Endogenous loading involves transfection to make donor cells express certain molecules or incubation of the donor cells with drugs, allowing the cells to uptake these drug molecules and subsequently secrete drug-loaded EVs^[109]^. Endogenous loading does not disrupt the structure and activity of EVs and provides stable loading; however, loading efficiency is relatively low^[110]^. Exogenous loading involves isolating EVs from cells and then loading drugs into the vesicles through physical or chemical methods such as electroporation, sonication, membrane permeabilization, freeze-thaw cycles, and extrusion^[111]^. Exogenous loading offers a higher loading efficiency but can affect the structure and activity of EVs to some extent^[112,113]^. The choice of drug-loading method depends on specific experimental needs, with detailed applications provided in Tables 4 and 5.

**Table 4:** Applications of endogenous loading strategies for EVs

**Cargo**	**Loading method**	**Isolation method**	**Loading efficiency**	**Ref.**
Oxaliplatin	Incubation	SEC	-	[114]
PEG-HGNs	Incubation	-	~ 50%	[115]
DSPE-PEG-RGD	Incubation	UC	-	[116]
Linezolid	Incubation	Precipitation	-	[117]
microRNA let-7a-5p	Transfection	UC, UF	The let-7a-5p copy numbers within 1 × 10^10^ EVs had 6.46 × 10^10^ copies	[118]
MicroRNA-27a	Transfection	UF	-	[119]
VMP1	Transfection	UF, UC	-	[120]
pDNA/CPP	Transfection	Precipitation, UF, SEC	76% to 86%	[121]
GDF-15	Transfection	UC, UF	-	[122]
RAGE siRNA	Transfection	UC	-	[123]
CD64^WT^, CD64^CK^, TP53, and small hairpin KRAS^G12D^	Transfection	UC, UF, SEC	The colocalization ratios of encapsulated TP53 mRNA or siKRAS^G12D^ and CD64^CK^ surface protein within dtEV were ~ 60%	[124]

EVs: Extracellular vesicles; PEG: polyethylene glycol; UC: ultracentrifugation; UF: ultrafiltration; SEC: size exclusion chromatography.

**Table 5:** Applications of exogenous loading strategies for EVs

**Cargo**	**Loading method**	**Isolation method**	**Loading efficiency**	**Ref.**
microRNA	Extrusion	UC, UF	-	[125]
Clodronate	Extrusion	UC	-	[126]
Dapagliflozin	Extrusion	UC	~ 45%	[127]
PTX	Extrusion	UC, UF	~ 14.23%	[128]
GQDs/Cy5-miR	Sonication	UC	5.6 times higher than the untreated sEVs	[129]
Erastin and Rose Bengal	Sonication	UC	-	[130]
PTX	Sonication	UC	-	[131]
Tripeptidyl peptidase-1	Sonication	UC	-	[132]
FX	Sonication	UC, UF	The loading rates of LpEVs-FX and GA-LpEVs-FX were approximately 69% and 86%	[133]
miRNA-497, DOX	Membrane Permeabilization	UC, UF	4.3-fold and 7.2-fold greater than traditional methods	[134]
miR-195-5p	Electroporation	UC	-	[135]
siRNA	Electroporation	UC	-	[136]
IL-10	Electroporation	UC	~ 70%	[137]
DDHD1-siRNA	Electroporation	UC	13%	[138]
CCL2-siRNA	Electroporation	UC	-	[139]
miR-223	Electroporation	UC	around 60%	[140]
Ca(HCO_3_)_2_	Electroporation	UC	-	[141]
Dox	Electroporation	UC	-	[142]
HAL	Electroporation	UC	~ 25.14%	[143]
Dox	Electroporation	SEC, UF	190-fold increased response compared to naked doxorubicin	[144]
gRNA: Cas9 RNP complexes	Electroporation	UC	~ 80%	[145]
Zinc oxide Nanocrystals	Freeze-Thaw	UC	-	[146]

EVs: Extracellular vesicles; UC: ultracentrifugation; UF: ultrafiltration; FX: fucoxanthin; IL-10: interleukin-10; RNP: ribonucleoprotein; SEC: size exclusion chromatography.

## MECHANISMS OF CANCER DRUG RESISTANCE

Drug resistance is a major challenge for effective cancer treatment. Depending on the treatment method, cancer resistance can be classified as chemotherapy, targeted therapy, immunotherapy, or endocrine therapy resistance. The mechanisms of cancer resistance include increased drug efflux, reduced drug uptake, drug inactivation, target mutations, signaling pathway alterations, apoptotic defects, phenotype switching, and resistance to immunotherapy.

The following sections introduce the specific mechanisms of cancer resistance and the methods to overcome them.

### Increased drug efflux

Drug efflux refers to the process by which cells expel drug molecules from the inside to the outside through specific transport proteins on the membrane. These transport proteins primarily belong to the ATP-binding cassette (ABC) transporter family, including P-glycoprotein (P-gp), multidrug resistance-associated proteins (MRPs), breast cancer resistance protein (BCRP), cholesterol transport protein (ABCA1), and bile salt export pump (BSEP). They regulate drug distribution, absorption, and excretion, thereby protecting cells from death caused by high intracellular drug concentrations^[1]^. During tumor treatment, ABC transporters can reduce the effective concentration of drugs inside tumor cells, resulting in multidrug resistance (MDR).

In most cases, overcoming the resistance caused by increased drug efflux involves increasing drug concentration; however, this often leads to systemic toxicity^[1]^. The latest strategy involves collateral sensitivity (CS), in which cells exhibiting resistant phenotypes owing to ABC transporter overexpression are highly sensitive to certain compounds. This phenomenon is known as CS and can be considered a lethal weakness in cancer cells overexpressing ABC transporters^[147]^. Zhang *et al.* reported that using BAY-1082439 and CRISPR/Cas9 to knock out *Pik3ca* and *Pik3cb* can inhibit the activation of PI3K 110α and 110β catalytic subunits, downregulating ABC transporters P-gp/ABCB1 and BCRP/ABCG2, thereby re-establishing drug sensitivity in resistant epidermoid carcinoma and non-small cell lung cancer (NSCLC) cells^[148]^. They also discovered that CDK6 deletion altered ABCB1 levels, increased drug accumulation in tumors, significantly inhibited tumor growth and metastasis, and improved survival. Thus, the CDK6-PI3K pathway may serve as a new target for reversing ABCB1-mediated cancer resistance^[149]^. Additionally, computer-aided drug design, high-throughput screening, or structure-based drug design and synthesis can be used to develop chemotherapeutic adjuvant inhibitors of ABC transporters^[150-153]^.

### Reduced drug uptake

Similar to increased drug efflux, reduced drug uptake decreases the concentration of drug molecules in the cellular environment, thereby limiting their efficacy. Chemotherapeutic drugs such as methotrexate, 5-fluorouracil, 8-azaguanine, and cisplatin are most affected by this resistance mechanism. They use transport proteins, such as solute carrier (SLC) transporters, to enter cells. Downregulation of SLC transporter expression and function in tumor cells limits drug uptake, leading to reduced drug efficacy^[154]^. Overcoming this resistance mechanism involves maximizing the passive permeability of anticancer drugs or using alternative active transport pathways^[3]^.

Nonselective upregulation of SLC transporters can lead to increased drug accumulation in normal cells. Therefore, it is crucial to develop drugs that selectively regulate the expression of SLC transporters in cancer cells^[155,156]^. Huttunen *et al.* showed that attacking several SLCs to alter the nutrient environment of cancer cells could serve as adjuvant therapy for other chemotherapeutic drugs, providing an alternative to ABC transporter inhibitors^[157]^. The poor prognosis of advanced metastatic differentiated thyroid cancer (DTC) is primarily due to reduced expression of the sodium/iodide symporter (NIS) or decreased targeting of NIS to the cell membrane, which reduces the efficacy of radioactive iodine therapy^[158]^. Ullmann *et al.* reported that dual treatment with BRAF and MEK inhibitors could upregulate NIS expression, increase iodine uptake in tumors, enhance efficacy, and overcome resistance^[159]^.

### Drug inactivation

After drugs enter the body and exert their effects, they are metabolized and excreted. Carrier molecules and enzymes responsible for drug metabolism play important roles in resistance to chemotherapy^[1]^. Glutathione (GSH) is involved in maintaining the cellular redox balance and can detoxify xenobiotics, enhancing MDR in cancer cells^[1]^. Reducing GSH levels is an effective strategy for increasing the sensitivity of cancer cells to chemotherapy^[160]^. Some drugs require enzymatic metabolism to become active, and mutations or downregulation of these enzymes can affect drug efficacy. For example, cytarabine (Ara-C) must be metabolized into the active substance Ara-C triphosphate. Mutations or downregulation of enzymes such as deoxycytidine kinase can lead to drug resistance in tumor cells^[161]^.

The cytochrome P450 (CYP) family primarily participates in drug metabolism. Overexpression of these enzymes has been observed in drug-resistant tumor cells. Kawahara *et al.* discovered that overexpression of the CYP enzymes CYP3A4 and CYP2C8 in malignant breast cells leads to the metabolic inactivation of paclitaxel in liver tissue^[162]^. Cytochrome P450 1B1 (CYP1B1) is overexpressed in various solid tumors. Hachey *et al.* used scaffold hopping to explore the chemical space of CYP1B1 inhibitors and found a new lead compound that inhibited CYP1B1 in trace amounts, potentially restoring drug sensitivity in resistant cells^[163]^.

### Target mutation

Target mutations can lead to the development of resistance to therapeutic molecules^[3]^. Target mutations include steric impact on drug binding, impact on cofactor affinity, and conformational change of target.

#### Steric impact on drug binding

First, the substitution of smaller amino acids with larger ones increases steric hindrance and reduces the affinity of the drug molecules. Second, mutations of polar amino acid side chains to hydrophobic ones, or vice versa, can disrupt polar or nonpolar interactions and increase steric hindrance^[3]^. Abnormal activation of CDK9 is associated with the super-enhancer-mediated transcription of short-lived proteins required for cancer cell survival. Targeting CDK9 results in potent antitumor activity. Hu et al. showed that tumor cells develop resistance to CDK9 inhibitors due to the CDK9 L156F mutation, which disrupts the binding of the inhibitor to CDK9 through steric hindrance, thereby affecting the stability and catalytic activity of the CDK9 protein^[164]^. Targeting the proto-oncogene protein RET with small-molecule tyrosine kinase inhibitors (TKIs) is an effective treatment strategy for thyroid cancer. Meng et al. reported that mutations in the gatekeeper residues V804M and V804L of the RET kinase domain create steric hindrance, leading to TKI resistance^[165]^.

#### Impact on cofactor affinity

If the mechanism of action of a drug involves competition with cofactors or substrates, target mutations that increase affinity for cofactors or substrates can lead to resistance^[3]^. Conversely, mutations that decrease drug-target affinity can also cause resistance. Neratinib, a HER2 tyrosine kinase inhibitor (TKI), is effective for the treatment of breast cancer. The HER3 E928G kinase domain mutation enhanced the affinity of HER2/HER3, reducing the binding of HER2 to neratinib, thereby causing resistance^[166]^.

#### Conformational change of target

Mutations can cause resistance by inducing conformational changes that activate kinase mechanisms^[3]^. Most HER2 exon 20 insertions (ex20ins) are resistant to EGFR or pan-HER TKIs. Zhao et al. showed that ex20ins in HER2-mutant lung cancers lead to ligand-independent kinase activation by altering the conformational landscape of HER2 kinase and restricting its conformation in the active state. This mechanism explains the TKI^[167]^.

### Signaling pathway alterations

Tumor cells can achieve resistance to drug treatment by activating certain signaling pathways through specific gene changes^[168]^.

BRAF inhibitors (BRAFis) are targeted drugs used to treat melanoma. Wang *et al*. showed that treatment with BRAFis increased the expression of IRF9 and STAT2 in melanoma cells. Overexpression of IRF9 or STAT2 slowed BRAFis-induced tumor shrinkage, while knockdown of IRF9 or STAT2 accelerated BRAFis-induced tumor shrinkage. IRF9-STAT2 signaling controls GSDME-dependent pyroptosis by restoring GSDME transcription. Targeting IRF9/STAT2 may prevent BRAFis resistance in melanoma by inducing pyroptosis^[169]^. Kobayashi *et al*. summarized the signaling pathways related to cancer immune evasion and immune checkpoint inhibitor (ICI) resistance^[170]^.

### Apoptotic defects

The goal of most cancer drugs is to induce tumor-selective cell death. Disruption of apoptotic mechanisms can lead to drug resistance, particularly in cases where chemotherapy resistance is related to defects in cell death mechanisms^[3]^.

The first-line treatment for most hematological malignancies (HMs) relies primarily on cytotoxic agents. However, HM recurrence is often associated with defects in the DNA damage response (DDR) pathways and anti-apoptotic blocks. BH3 mimetics represent a new class of pro-apoptotic anticancer drugs that can directly target the mitochondria, independent of TP53 gene aberrations. These drugs effectively clear non-dividing malignant cells with adverse molecular cytogenetic alterations^[171]^. Treatment of prostate cancer (PCa) primarily inhibits tumor growth by inducing apoptosis. However, defects in the apoptotic response frequently lead to resistance. Therefore, triggering non-apoptotic cell death may be an alternative strategy for preventing cancer resistance. Montagnani Marelli *et al.* found that the combination of δ-tocotrienol (δ-TT) and docetaxel (DTX) enhanced DTX cytotoxicity in DU145 cells. In addition, δ-TT induced necrotic apoptosis in DTX-resistant cells^[172]^.

### Phenotype switching

Phenotype switching refers to the ability of cells to change among various forms, allowing them to adapt to environmental changes. This switching can serve as a drug resistance mechanism independent of drug-target pathways. The molecular mechanisms underlying cancer cell phenotype remodeling include both endogenous and exogenous factors. Endogenous mechanisms involve changes in epigenetic modifications and the expression of related transcription factors, whereas exogenous mechanisms are primarily related to changes in the tumor microenvironment (TME), such as hypoxia, infiltration of tumor-promoting immune cells, and the release of pro-inflammatory factors^[173]^.

The phenotypic transition from EGFR-mutant lung adenocarcinoma (LUAD) to small cell lung cancer (SCLC) is considered a clinical mechanism of resistance to EGFR inhibitors^[3]^. It has been reported that EHMT2 participates in SCLC transformation and EGFR-TKI resistance through enzyme activity-dependent mechanisms^[174]^. EHMT2 affects the accumulation of H3K9me2 in the promoter region of SFRP1, thereby remodeling chromatin structure and inhibiting the expression of the WNT/β-catenin pathway negative regulator SFRP1. This abnormal activation of the WNT/β-catenin pathway in SCLC-transformed cells ultimately drives the neuroendocrine transformation of NSCLC to SCLC.

### Immunotherapy resistance

The mechanisms of resistance to cancer immunotherapy involve interactions among tumor cells, the TME, and the host immune system. These resistance mechanisms can be divided into several major categories:

#### Tumor Cell Evasion

Initially, tumor cells overexpress immune checkpoint molecules such as PD-L1 (programmed death-ligand 1), TIM-3 (T-cell immunoglobulin and mucin-domain containing-3), LAG-3 (lymphocyte-activation gene 3), and VISTA (V-domain Ig suppressor of T cell activation), which inhibit T cell activation by binding to their respective receptors^[175]^. Concurrently, genetic mutations, like those in the β-2 microglobulin (B2M) gene, reduce MHC-I expression, preventing effective antigen presentation and T cell recognition^[176,177]^. To ensure survival once recognized, tumor cells upregulate anti-apoptotic proteins such as BCL-2, making them resistant to immune-mediated apoptosis^[178]^. Additionally, tumor cells leverage epigenetic modifications - such as DNA methylation - to regulate tumor antigens and immune-related gene expression, dynamically adapting to immune pressures^[179]^. Furthermore, tumor cell plasticity, including epithelial-mesenchymal transition (EMT), allows them to alter their phenotype to evade immune attack, and tumor heterogeneity creates distinct subpopulations, complicating treatment due to differential responses to therapy^[180]^. Together, these mechanisms form a multi-layered defense strategy that enables tumor cells to escape immune detection, resist treatment, and ultimately contribute to therapeutic failure.

#### Abnormal Tumor Vasculature

Tumor vessels are often irregular and tortuous, resulting in unstable blood flow and poor perfusion, effectively limiting immune cell infiltration^[181]^. Additionally, the abnormal structure of tumor vasculature leads to hypoxia, which suppresses effector immune cell activity and drives the production of immunosuppressive factors such as VEGF, IL-10 (interleukin-10), and TGF-β (transforming growth factor-beta), while also promoting PD-L1 upregulation, thereby facilitating immune evasion^[182]^. Furthermore, plasma leakage from these abnormal vessels creates high interstitial pressure, forming a physical barrier that further hinders immune cell migration and reduces immunotherapy effectiveness^[183]^.

#### Immunosuppressive TME

The TME often contains a high concentration of immunosuppressive cells, such as regulatory T cells (Tregs), myeloid-derived suppressor cells (MDSCs), and tumor-associated macrophages (TAMs), which secrete inhibitory cytokines (e.g., TGF-β and IL-10)^[184]^. Moreover, metabolic products like lactate and adenosine accumulate in the TME, further inhibiting immune cell activity, while hypoxia promotes PD-L1 expression, reducing the immune system’s ability to recognize and attack tumor cells^[185]^.

#### T Cell Dysfunction

T cells can become exhausted after prolonged antigen exposure, characterized by the overexpression of inhibitory receptors and decreased secretion of effector cytokines. Additionally, some tumors inhibit the formation of memory T cells, increasing the likelihood of relapse after initial treatment^[186,187]^.

## ENGINEERING EVS AS DRUG CARRIERS TO OVERCOME DRUG RESISTANCE

Drug resistance is closely related to tumor recurrence and mortality, posing the greatest obstacle to clinical cancer treatment. Therefore, developing effective strategies to overcome cancer resistance is crucial. This section focuses on the progress in the use of EVs as drug carriers to overcome resistance to cancer treatments, including chemotherapy, targeted therapy, immunotherapy, and endocrine therapy.

### Overcoming chemotherapy resistance

Studies have shown that EVs can improve the efficacy of chemotherapy by delivering chemotherapeutic drugs, RNA, or other biomolecules to regulate cancer chemotherapy resistance [Table 6].

**Table 6:** EVs in overcoming cancer chemotherapy resistance

**EVs source**	**Cargo carried**	**Drug**	**Mechanisms**	**Ref.**
Melanoma cells	survivin-T34A	Gemcitabine	Block Survivin, induce caspase activation and apoptosis	[188]
AMSCs	miR-199a	DOX	Inhibit mTOR activation and the phosphorylation of 4EBP1 and 70S6K	[189]
BM-MSCs	miR-124	5-FU	Suppress cell viability, invasion, and migration, as well as inducing cell apoptosis in pancreatic cancer by regulating EZH2	[190]
MSCs	miR-1236	DDP	SLC9A1 downregulation and Wnt/β-catenin inactivation	[191]
hUCMSCs	miR-146a	DTX, TAX	Reduce LAMC2 expression via the PI3K/AKT signaling pathway	[192]
BMSCs	miR-193a	DDP	Suppress the colony formation, invasion, migration, and proliferation, as well as advancing apoptosis by downregulating LRRC1	[193]
MSCs	anti-miR-9	TMZ	Decrease the expression of the drug transporter gene MDR1	[194]
HEK293T cells	lncRNA DARS-AS1 siRNA	DOX	Suppress TGF-β/Smad3 signaling pathway-induced autophagy	[18]
hMSCs	miR-199a	TMZ	Downregulation of AGAP2	[195]
WJ-MSCs	miR-124	TMZ	-	[196]
HEk293T cells	si-c-Met	Cisplatin	Si-c-Met inhibited migration, invasion and promoted apoptosis *in vitro*	[20]
MDA-MA-231, MCF-7, 4T1 cells	siMTA1	Gemcitabine	Enhance the sensitivity of MCF-7 to gemcitabine and weaken the metastasis ability of MCF-7	[197]
HCT116, SW480 cells	CPT1A siRNA	Oxaliplatin	Silence CPT1A by siRNA or etomoxir, a specific small-molecule inhibitor of CPT1A	[198]
TMZ-resistant cells	miR-151a	TMZ	Restore miR-151a expression sensitized TMZ-resistant GBM cells via inhibiting XRCC4-mediated DNA repair	[199]
HEK293T, HUVEC, OVCAR-3 cells	miR-484	Cisplatin	induce vessel normalization	[200]
HEK293T cells	anti-miR-214	Cisplatin	Downregulate miR-214 and overexpress possible target proteins	[201]
HEK293T cells	miR-374a-5p inhibitor	Oxaliplatin	Inhibit MiR-374a-5p	[202]
UPCI-SCC-131 cells	miR-155 inhibitor	Cisplatin	Upregulate FOXO3a and inhibit drug efflux transporter expression	[203]
4T1 cells	anti-miR-21	DOX	Block the function of endogenous carcinogenic miR-21	[204]
293T cells	PGM5-AS1	Oxaliplatin	Prevent proliferation, migration, and acquired oxaliplatin tolerance	[205]
FHCs	circ-FBXW7	Oxaliplatin	Exosome mediated circ-FBXW7 transfer enhances oxaliplatin sensitivity by binding to miR-18b-5p	[206]
BM-MSCs	PTX, GEMP	Paclitaxel, Gemcitabine	Overcome the restrictions of pathological ECM and enhance the accumulation of therapeutics in tumor	[207]
Mononuclear M1 and M2 macrophages	Cisplatin	Cisplatin	Chemotherapy drugs preferentially accumulate in cancer cells and M1 macrophages have the effect of killing tumor	[208]
SKOV3-CDDP cells	miR497、TP	Cisplatin	Inhibit the PI3K/AKT/mTOR signaling pathway, deplete GSH, elevate intracellular ROS, and regulate macrophage polarization	[209]
HEK293T cells	DOX	DOX	Reduce P-gp expression in drug-resistant MCF7/ADR cells	[210]
Fibroblasts	CD47 GM-CSF DTX	DTX	Improve drug delivery efficiency and enhance the immune response in the tumor microenvironment	[211]
THLG-293T, LG-293T cells	5-FU, miR-21i	5-FU	Induce cell cycle arrest, reduce tumor proliferation, increase apoptosis, inhibit the tumor cell migration, and suppress PTEN and hMSH2 expressions	[212]
RAW 264.7 macrophages	PTX	PTX	Increase PTX solubility and overcome P-gp-mediated drug efflux	[213]

EVs: Extracellular vesicles; P-gp: P-glycoprotein; MSCs: mesenchymal stem cells.

#### Overcoming drug efflux-induced resistance

miR-9 is upregulated in temozolomide (TMZ)-resistant Glioblastoma (GBM) cells and is involved in the expression of the drug efflux transporter P-gp. Munoz *et al*. loaded anti-miR-9 into EVs derived from mesenchymal stem cells (MSCs), thereby reducing the expression of proteins associated with resistance^[194]^. Liu *et al*. inserted lipidomimetic chain-conjugated HA (lipHA) into the membranes of EVs^[210]^. As lipHA-hEVs can reduce P-gp expression in resistant MCF7/ADR cells, loading doxorubicin onto lipHA-hEVs effectively inhibited its efflux, thus overcoming doxorubicin resistance^[210]^. Treatment of metastatic colorectal cancer (mCRC) is inevitably affected by oxaliplatin resistance. Studies have shown that EV-delivered circular RNA F-box and WD repeat domain-containing 7 (circ-FBXW7) can bind to miR-128-5p and restore oxaliplatin sensitivity in resistant CRC cells by inhibiting drug efflux, thus providing a novel treatment strategy for patients with CRC with oxaliplatin resistance^[206]^.

#### Overcoming reduced drug uptake-induced resistance

Studies have shown that loading cisplatin onto EVs derived from mononuclear M1 and M2 macrophages results in the preferential accumulation of the chemotherapeutic drug in tumors, significantly increasing its cytotoxicity against resistant A2780/DDP and A2780 cells^[208]^. Hyperthermic intraperitoneal chemotherapy (HIPEC) is a standard clinical treatment for metastatic peritoneal cancer. However, its efficacy is limited by low drug penetration efficiency and the rapid development of resistance. Lv *et al*. prepared genetically engineered exosome-thermosensitive liposome hybrid NPs (gETL NPs) to enhance drug delivery efficiency and overcome drug resistance^[211]^. gETL NPs effectively penetrated tumor tissue after intravenous injection and released DTX under the low-temperature conditions of HIPEC, increasing drug concentration and efficacy in tumors. In addition, CD47 molecules on the surface of gETL NPs bound to the signal regulatory protein alpha receptor (SIRP𝛼) on macrophages, preventing tumor cells from escaping the immune system, thus enhancing macrophage phagocytosis of tumor cells. Furthermore, gETL NPs delivered GM-CSF (granulocyte-macrophage colony-stimulating factor), promoting the polarization of macrophages from the M2 type to the M1 type, further enhancing antitumor capability. By improving drug delivery efficiency and enhancing the immune response in the TME, gETL NPs effectively inhibited tumor growth and reduced resistance.

#### Overcoming drug inactivation-induced resistance

The primary reasons for gemcitabine resistance in pancreatic ductal adenocarcinoma (PDAC) are decreased activity of gemcitabine metabolism-limiting enzymes and insufficient conversion of gemcitabine phosphate products. Studies have shown that loading PTX, gemcitabine monophosphate (GEMP), and the intermediate products of gemcitabine metabolism onto EVs can overcome chemotherapy resistance^[207]^.

#### Overcoming signal pathway alteration-induced resistance

Studies have shown that EVs can deliver miR-199a to target the mTOR pathway, inhibiting mTOR activation and subsequent phosphorylation of 4EBP1 and 70S6K in hepatocellular carcinoma (HCC) cells, thus restoring doxorubicin sensitivity in HCC cells^[189]^. Li *et al.* fused EVs from SKOV3-CDDP cells with liposomes modified with the tumor-targeting peptide cRGD and loaded them with triptolide (TP) and miR-497 to create miR-497/TP-HENPs^[209]^. These miR-497/TP-HENPs can overcome cisplatin resistance in tumor cells by inhibiting the PI3K/AKT/mTOR signaling pathway, increasing reactive oxygen species (ROS) production, and regulating macrophage polarization.

#### Overcoming apoptosis defect-induced resistance

GBM is a common primary malignant brain tumor in adults, and TMZ is the only chemotherapeutic drug with clear efficacy against GBM. However, TMZ resistance severely affects treatment outcomes. GBM resistance to TMZ is associated with the loss of miR-151a. Engineered EVs carrying miR-151a inhibited XRCC4-mediated DNA damage repair and restored TMZ sensitivity in GBM^[199]^. Defects in apoptosis in NSCLC lead to cisplatin resistance, which is an important cause of clinical treatment failure. Studies have shown that miR-193a promotes apoptosis in cisplatin-resistant NSCLC cells by downregulating LRRC1 and can also inhibit colony formation, invasion, proliferation, and migration. Wu *et al*. have developed EVs derived from human BM-MSCs loaded with miR-193a to reduce NSCLC resistance to cisplatin treatment^[193]^. In addition, the sensitivity of gastric cancer cells to cisplatin is closely linked to c-Met siRNA, with resistant cells exhibiting lower c-Met siRNA levels compared to sensitive cells. Researchers have used EVs derived from HEK293T cells to deliver c-Met siRNA, thereby enhancing the sensitivity of cisplatin-resistant gastric cancer cells by promoting apoptosis^[20]^. The apoptosis inhibitor protein survivin is considered an important factor in resistance to various cancer treatments. Using melanoma-derived EVs to deliver Survivin-T34A significantly induces caspase activation, enhances the cytotoxic effect of gemcitabine, and promotes pancreatic cancer cell apoptosis^[188]^.

### Overcoming targeted therapy resistance

Targeted antitumor drugs act specifically on tumor cells, minimizing effects on normal cells. This precision improves efficacy and reduces adverse reactions. However, long-term use of these drugs may lead to resistance. These mechanisms include genetically related resistance due to target mutations and non-genetic resistance due to the phenotypic remodeling of tumor cells under the influence of epigenetic factors or transcriptional regulation. EVs may be a new method and strategy for overcoming resistance to targeted therapy [Table 7].

**Table 7:** EVs in overcoming resistance to other therapies

**Treatment**	**EVs source**	**Cargo carried**	**Drug**	**Mechanisms**	**Ref.**
Targeted therapy	HEK293T cells	BCR-ABL siRNA	IM	Inhibits BCR-ABL and improves the combination of the imatinib	[214]
	hUC-MSCs	-	IM	Promotes cell viability inhibition and apoptosis and increases Bax expression and decreases Bcl-2 expression	[215]
	AMSCs	miR-122	Sorafenib	Downregulates the expression of *CCNG1*, *IGF1R*, and *ADAM10* genes, and upregulates the expression of *Caspase 3* and Bax	[19]
	BM-MSCs	siGRP78	Sorafenib	Inhibits the expression of GRP78 in sorafenib-resistant cancer cells	[216]
	HCC cells	siRNA-1	Sorafenib	Prevents PRP19-mediated YBX1 ubiquitination and degradation in the nucleus	[217]
Immunotherapy	B16F10 cells	POM1, Metformin	PD-1	Increases the level of pro-inflammatory extracellular ATP to prevent the accumulation of immunosuppressive adenosine and alleviates hypoxia	[218]
	BM-MSCs	Galectin-9 siRNA	-	Blocks galectin-9/dectin-1 axis to reverse immunosuppression	[219]
Endocrinotherapy	HUCMSCs	miR-let-7c	-	Antagonizes androgen receptor expression and activity by downregulating HMGA2, RAS, and Myc	[220]

EVs: Extracellular vesicles; MSCs: mesenchymal stem cells; HCC: hepatocellular carcinoma.

#### Overcoming target mutation-induced resistance

Imatinib (IM) is a selective Bcr-Abl inhibitor, and mutations in the *BCR-ABL* gene reduce its binding affinity for IM, resulting in acquired resistance. Bellavia *et al.* prepared EVs that can deliver BCR-ABL siRNA to CML cells and overcome targeted drug resistance by inhibiting Bcr-Abl^[214]^.

#### Overcoming pathway alteration-induced resistance

circRNA-SORE is upregulated in sorafenib-resistant HCC cells. This prevents PRP19-mediated YBX1 degradation, affects the expression of downstream YBX1 target genes, and ultimately leads to sorafenib resistance. Tumor-derived exosomes delivering si-circRNA-SORE significantly increased the sensitivity of tumor cells to sorafenib treatment^[217]^. Sorafenib-resistant cancer cells overexpress GRP78 to a greater extent than sorafenib-sensitive cancer cells, making GRP78 a therapeutic target for HCC^[221]^. Li *et al*. prepared exosomes loaded with siGRP78 and observed that they inhibited GRP78 expression in sorafenib-resistant cancer cells. Therefore, the combination of these exosomes with sorafenib enhanced the inhibition of HCC cell growth and metastasis *in vivo*^[216]^. Studies have shown that EVs transfected with miR-122 reduce the resistance of HCC cells to sorafenib. This may be because miR-122-loaded EVs reduce the expression of *CCNG1*, *IGF1R*, and *ADAM10* genes, upregulate the expression of the apoptosis-related genes *Caspase 3* and Bcl-2-associated X protein (*Bax*), and increase HCC cell sensitivity to sorafenib^[19]^.

### Overcoming immunotherapy resistance

The advent of immunotherapy has brought cancer treatment into a new era^[222]^. However, the resulting resistance has also led to most patients with cancer not benefiting clinically. Researchers have used exosomes to overcome resistance to immunotherapy [Table 7].

Wu *et al*. used EVs derived from B16F10 cells to deliver POM1 (a CD39 antagonist) and metformin (an AMPK agonist) for cancer treatment. They demonstrated that this platform elevated extracellular ATP (eATP) levels, which triggered the activation of the P2X7-NLRP3-inflammasome to drive macrophage pyroptosis and potentiated the maturation and antigen capacity of DCs. This, in turn, enhances the cytotoxic function of T cells and NK cells, leading to a synergistic antitumor immune response that suppresses cancer progression, metastasis, and recurrence while overcoming anti-PD1 resistance^[218]^. A previous study showed that CD38 is highly expressed in HCC, and EVs/siCD38 inhibit CD38 enzyme activity, decrease adenosine production, and promote macrophage repolarization to the M1 type, thereby inhibiting HCC cell growth and metastasis *in vitro* and tumor growth in mice and overcoming PD-1/PD-L1 inhibitor resistance^[223]^.

### Overcoming endocrine therapy resistance

Endocrine therapy, also known as hormone therapy, is a method for treating cancer by slowing or stopping the growth of certain hormone-dependent cancer tissues. The most commonly targeted pathways are the estrogen and androgen signaling pathways, which play key roles in breast and prostate cancers, respectively^[224]^. The growth of PCa cells depends on androgens. Therefore, removing or inhibiting androgen activity, known as androgen deprivation therapy (ADT), is the primary approach to treating PCa. However, patients undergoing ADT eventually transition from castration-sensitive to castration-resistant PCa, leading to ADT resistance. Kurniawati *et al.* used MSCs loaded with lethal 7c (let-7c), which antagonizes androgen receptor (AR) expression and activity by downregulating HMGA2, RAS, and Myc, thereby overcoming ADT resistance^[220]^.

## CONCLUSION

EV-based drug delivery systems are promising and multifaceted for overcoming cancer drug resistance. As advancements in EV engineering and functionalization continue, these systems have the potential to enhance the precision and efficacy of cancer therapies by specifically targeting resistant cancer cells, thereby minimizing off-target effects and systemic toxicities. Integrating EV-based delivery with existing therapies can create synergistic effects and simultaneously overcome multiple resistance mechanisms. The unique ability of EVs to cross biological barriers such as the blood-brain barrier further expands their therapeutic potential, particularly for metastatic and hard-to-reach tumors. Continued improvements in EV isolation, characterization, and functionalization will enhance their therapeutic capabilities.

Despite the considerable potential of EV therapy in cancer treatment, its practical application faces significant obstacles. The high heterogeneity of EVs complicates standardization, as EVs from different sources vary widely in size, composition, and function, impairing efficacy assessment and reproducibility. Additionally, the lack of industrial standards for purification and stabilization remains a major barrier, with existing isolation methods being inefficient, costly, and unable to guarantee high purity or consistency. Moreover, verifying the therapeutic efficacy of EVs is still a big challenge due to the complex *in vivo* environment, which involves biological barriers, making precise control over drug delivery and efficacy evaluation difficult.

Nevertheless, ongoing technological advancements and research efforts present viable opportunities to overcome these challenges in EV therapy. Establishing standardized processes for EV preparation and purification, refining engineering modifications, and gaining deeper insights into *in vivo* delivery mechanisms are essential steps to improve the efficacy and reproducibility of EV-based treatments. In addition, harnessing the synergistic potential of EVs in combination with established therapies, such as immunotherapy and chemotherapy, may offer promising solutions to overcome cancer resistance. Although EV therapy holds substantial promise as a safe, effective, and personalized cancer treatment, its successful clinical application still needs significant research support and the establishment of appropriate regulatory standards.

## DECLARATIONS

### Copyright

© The Author(s) 2024.
